# Three machine learning models for the 2019 Solubility Challenge

**DOI:** 10.5599/admet.835

**Published:** 2020-06-15

**Authors:** John B. O. Mitchell

**Affiliations:** EaStCHEM School of Chemistry and Biomedical Sciences Research Complex, University of St Andrews, North Haugh, St Andrews, Scotland, KY16 9ST, UK

**Keywords:** Aqueous intrinsic solubility, Solubility prediction, Random Forest, Extra Trees, Bagging, Consensus classifiers, Wisdom of Crowds, Inter-laboratory error

## Abstract

We describe three machine learning models submitted to the 2019 Solubility Challenge. All are founded on tree-like classifiers, with one model being based on Random Forest and another on the related Extra Trees algorithm. The third model is a consensus predictor combining the former two with a Bagging classifier. We call this consensus classifier Vox Machinarum, and here discuss how it benefits from the Wisdom of Crowds. On the first 2019 Solubility Challenge test set of 100 low-variance intrinsic aqueous solubilities, Extra Trees is our best classifier. One the other, a high-variance set of 32 molecules, we find that Vox Machinarum and Random Forest both perform a little better than Extra Trees, and almost equally to one another. We also compare the gold standard solubilities from the 2019 Solubility Challenge with a set of literature-based solubilities for most of the same compounds.

## Introduction

Aqueous solubility remains one of the most significant challenges in drug development, with failure to produce bioavailable compounds potentially denying patients much-needed therapeutic interventions, while costing pharmaceutical companies years of time and hundreds of millions of dollars, euros or pounds. The oft-quoted facts are that as many as 70% of drugs in development have problematic solubility issues [1], and inadequate water solubility remains a major cause of failure of drug development projects [2].

Solubility is also a significant challenge for computational chemistry [3, 4]. First principles approaches have made some progress in recent years, and in the longer term may provide the most satisfactory means of computing solubility [5-11]. However, currently such first principles methods require a substantial amount of computer time and, despite potentially providing more theoretical insight, are generally less accurate in their quantitative predictions [3] than are the more empirical informatics approaches.

Data-driven approaches have been developed over the years. Originally usually labelled QSPR (Quantitative Structure-Property Relationship), they became informatics, and more recently Machine Learning (ML). This reflects the use of more sophisticated computational algorithms to derive predictions of unknown solubilities from available experimental solubility data for similar compounds. Early efforts to predict solubility were simple linear regressions [12-14], these were followed by multi-linear regressions [15-17], and then by ML algorithms adopted from computer science and offering sufficient flexibility to model non-linear relationships. These include Artificial Neural Networks (ANN) [18], Support Vector Machines (SVM) [19], k-Nearest Neighbours (kNN) [20], Random Forest (RF) [21], and Deep Learning [22].

The cited publications and others in the field have trained and tested their models using a variety of different datasets. This makes comparison between different methodologies somewhat problematic. Thus, in 2008 the *Journal of Chemical Information and Modeling* announced a Solubility Challenge [23], with entrants invited to predict the newly measured and unrevealed intrinsic aqueous solubilities of 32 test compounds, given a training set of 100 values for a chemically similar druglike set. All 132 solubilities were measured using the CheqSol method [24]. The results, announced in 2009 [25] gave some insight into the then state of the field, although it would have been helpful to have learned rather more about the methodologies used by the 99 entrants.

The 2019 Solubility Challenge [26] has provided an opportunity to revisit this exercise, a decade on. This new challenge differed from its predecessor in a number of ways. There were two test sets provided, based on inter-laboratory averages of shake flask data, but no standardised training set. A first 100-compound test set was composed of tight low-variability data with inter-laboratory standard deviation given as ~0.17 log*S* units. Here and throughout this work, the base ten logarithm is used. A second 32-molecule set was listed as loose data with a higher reported inter-laboratory standard deviation of ~0.62 log*S* units. These sets contained numerous compounds whose solubilities have previously been reported in the literature, so it was left up to the entrants’ integrity and diligence to ensure that those compounds were omitted from whatever training data were used. A researcher was permitted to submit three predictions for each of the 2019 Solubility Challenge test datasets.

## Methods

### Data

A dataset of druglike organic compounds of known intrinsic aqueous solubility was prepared from the following sources: DLS-100 [27-29], 2008 Solubility Challenge [23, 25], Bergström *et al.* (2004) [30], and Wassvik *et al.* (2006) [31]. This dataset was constructed on the principle of one trustworthy high-quality measurement per compound, with CheqSol [24] measurements preferred where available, and shake flask data taken as the next preference. This differs from the construction of the test datasets in the 2019 Solubility Challenge, which were compiled on the basis of inter-laboratory average values. In total, the original set contained 205 solubility data points. Comparison of our data with the published compositions of the 2019 Solubility Challenge test sets revealed 52 compounds in common. Their removal left 153 compounds, which were divided into a training set of 117 molecules and an internal validation set of 36 compounds. The internal validation set was to be used for model parametrisation and model selection. The models were subsequently to be tested on the 100 compound low-variance tight test set and the 32 compound high-variance loose test set of the 2019 Solubility Challenge [26], these sets are respectively detailed in Tables A1 and A2 (Appendix).

### Machine Learning models

A number of machine learning methods were used in this work, all being implemented in the R programming language [32].

#### Random Forest

Random Forest [33, 34] is, as the name suggests, an ensemble of decision tree predictors. The individual trees are designed to be sufficiently stochastically different from one another for the resulting forest to benefit from the so-called wisdom of crowds, whereby a set of individually weak predictors can collectively function as a much stronger predictor [35, 36]. Each tree is built from a sample of *N* out of the *N* available data items, but chosen with replacement such that items may appear multiple times, once, or not at all in the dataset used for building a given tree. A further source of randomness is that only a limited selection of the possible features are made available to define the split at each node of the tree; with the selected split being chosen to be optimal amongst those available. For this study, we used the randomForest package in R [37]. Here, the randomForest routine was run to build 1000 trees, with default values of all other parameters.

#### Bagging

Bagging [38] follows the above description of Random Forest, except that *all* features are available for splitting at each node of the tree. Hence it is less random and more orientated towards use of individually more powerful features than Random Forest. The individual trees produced by bagging will be more mutually similar than those in Random Forest. The implementation of Bagging in the ipred package in R was used, building 100 trees.

#### Extra Trees

Extra Trees (Extremely Randomized Trees, ET), is a variant of Random Forest that differs in the following ways. Firstly, the original sample of *N* items is used for tree building, with no selection process. Secondly, the split at each node, while chosen using a random subset of features, is not fully optimised. Instead, one random cut-off point is selected for each descriptor, with subsequent optimisation limited to choosing amongst these partitions [39]. The implementation in the extraTrees R package was used for this solubility prediction project. Default values of all parameters in the extraTrees package were used, thus 500 trees were created.

#### Relevance Vector Machine

Relevance Vector Machine (RVM) [40] is a Bayesian kernel-based method often used for classification, but adapted also for regression. It has close similarities to both Support Vector Machine (SVM) and Gaussian Process algorithms. Compared to SVM, instead of support vectors RVM uses relevance vectors – based on typical representative members of each class. For regression problems such as this, the classification boundary is reimagined as a regression line, or hyperplane. We used the implementation in the kernlab package in R with the radial basis function kernel and the number of iterations set to 100.

#### k-Nearest Neighbours

k-Nearest Neighbours (kNN) is perhaps the simplest of all ML algorithms. Its predictions are based on the distances between a query item in the test set and its near neighbours in the training set. Distances are calculated in the feature space, which requires that descriptors should be scaled such that each dimension of the chemical space contributes fairly to the computed distances. For regression, the prediction for a given query item is based on the average property value (solubility) of its k closest neighbours in the training set’s scaled feature space. Thus if k = 4, the simplest kNN algorithm returns the average of the log *S* values of the four closest training compounds to the query. The contributions can alternatively be biased towards closer neighbours if an exponential distance-based weighting scheme is used [20], rather than a simple mean. For this project, we looked at both simple and exponentially weighted versions of kNN, and in each case at measures based on either Euclidean or Manhattan distances. Thus, we trialled four different variants of the kNN algorithm, and considered values of the parameter k from k = 2 to k = 8. For each of these four variants, the optimum value of k was determined by leave-one-out cross-validation (LOOCV) within the training set. Each of the four variants was run on the internal validation set with its own optimised k. Implementation of all four kNN variants was *via* the KernelKnn package in R [32, 41].

#### Multilayer Perceptron

The multilayer perceptron (MLP) is a feed-forward neural network, of a kind which we previously found to be the most effective single ML method in an earlier solubility prediction study using the DLS-100 dataset [28, 29]. We used the RSNNS package in R [32, 42], with descriptors scaled onto the range zero to one as for the kNN methodology. For MLP only, we similarly scaled the log *S* values onto the range between zero and one.

#### Vox Machinarum

We have already observed that ensembles of predictors benefit from a wisdom of crowds effect [35, 36], with a number of weaker predictors being combined to form a stronger predictive model. We used this idea to construct a consensus of ML models in Boobier *et al.* [28], which we compared in that paper with a Galton-style consensus of human predictors. Here, such a consensus ML model is given the name *Vox Machinarum*, chosen to echo the title of Galton’s paper *Vox Populi*. [35] The Vox Machinarum (VM) model consists of the median of some number of ML predictions for each test compound. The choice and number of ML predictors used are optimised using the internal validation set. Possible definitions using three, five, seven and nine other machine learning predictions for each compound were considered, each of these being implemented on the test set.

### Descriptors

We calculated CDK descriptors [43] for the training set, internal validation set, and for both 2019 Solubility Challenge test sets. Any descriptor that had an undefined value for any compound was removed from the set, as were all zero variance features. This resulted in a total of 173 usable descriptors for each compound. We used the randomForest R package [37] to assess the importance of each descriptor based on their individual effects on mean squared error for out-of-bag predictions of the training set, and on node purity. We also rank the descriptors according to their *R*^2^ measure of correlation with the training set log *S* values. The most important descriptors are shown in Table A3.

For the ensembles of tree-like models (Bagging, Random Forest and Extra Trees), we used all 173 descriptors, since these methods are considered robust to redundant information [34]. However, for the four kNN variants, for MLP and for RVM we carried out both selection and scaling on the features. We removed one of any pair of descriptors whose correlation coefficient had an absolute value > 0.8. We retained the descriptor with a higher absolute correlation coefficient with log *S* over the training set. Thus, we reduced the dimensionality substantially – the resulting chemical space was defined by 35 remaining descriptors. These remained non-orthogonal, so 35 is an overestimate of the true dimensionality of our chemical space. Each descriptor value was scaled to:



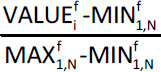



where VALUE^f^_i_ is the value of feature f for compound i, MAX^f^_1,_*_N_* is the maximum value that feature f takes for any of compounds 1 to *N*, and MIN^f^_1,N_ is the minimum value of f for any of compounds 1 to *N*. This ensures that all training set descriptors take a value in the range from zero to one. Test and internal validation set features are scaled using the MAX and MIN values from the training set to ensure that no test set data leak into the model construction process. For the MLP model only, log *S* values were scaled onto the range zero to one in the same way.

## Results and discussion

Results obtained on the 36-molecule internal validation set are summarised in Table 1. Here, RMSE is the Root Mean Squared Error of the predicted log *S* over the 36 compounds. AAE is the Average Absolute Error, *R*^2^ is the square of the Pearson Correlation Coefficient (not the coefficient of determination). The final two columns give the numbers of compounds, out of 36, with log *S* predicted to within 0.5 and within 1.0 log *S* units, respectively. Results are shown in Table 1 in order of increasing RMSE for each of nine individual machine learning methods, with the four kNN variants each being implemented with the k value pre-selected by LOOCV over the training set; that is k = 4 for the weighted and unweighted Manhattan distance-based kNN models, and k = 6 for the corresponding Euclidean distance-based models. Below this, the results for different possible definitions of Vox Machinarum, respectively using the median values from the best 3, 5, 7 or 9 predictors, are given also by ascending RMSE.

The rules of the Challenge stipulated that each entrant could submit only three models. Based upon these results, the three-predictor median version of Vox Machinarum, Extra Trees, and Random Forest were the selected models going forward to the actual Solubility Challenge. The third predictor chosen to contribute to the Vox Machinarum consensus was Bagging, which obtained the third best individual RMSE here.

### 2019 Solubility Challenge

The Extra Trees, Random Forest and Bagging classifiers were applied to each of the tight 100-compound test set and the tight 32-compound set provided by the organisers of the 2019 Solubility Challenge. These compounds were provided in the form of names and SMILES [44] codes. Several of the aromatic compounds in the test sets had SMILES codes which expressed these rings as alternating single and double bonds. Given that our training set molecules had been built with explicitly aromatic SMILES where relevant, at least for the substantial majority of cases, it was felt that consistency was needed between training and test sets. Test set SMILES were aromatised where appropriate by replacing alternating single-double bond SMILES codes with alternative SMILES containing explicit aromatic bonds; these changes affected 44/100 and 8/32 compounds in the respective test sets. Once these changes had been made, the same CDK descriptors as detailed above were calculated for all 132 test compounds. Given that all those methods requiring descriptor scaling had now been eliminated from consideration, no descriptor scaling or feature selection were performed. The same trained versions of the classifiers were used here as tested on the internal validation set, models that had already been trained on the original 117-compound training set.

The Random Forest, Extra Trees, and Bagging predictors were used to predict log *S* for each of the compounds in the sets of 100 and 32 molecules. Bagging predictions were not used in their own right, but only to contribute to the Vox Machinarum predictor. For each compound, three predictions were obtained, one from each of the three tree-based classifiers. The median of these three predicted values was used as the Vox Machinarum prediction for that compound. Three sets of (100 + 32) predictions were then submitted to the 2019 Solubility Challenge, one each for Vox Machinarum, Extra Trees, and Random Forest. These sets of predictions are given in full in Tables A1 & A2.

### 2019 Solubility Challenge: self-assessment on (89 + 26) compounds

After these three entries had been submitted to the 2019 Solubility Challenge, a self-assessment exercise was carried out. This required sourcing a single literature solubility for as many as possible of the 132 test compounds. In addition to the 52 values for test compounds that we had previously excluded from our training set, a further 63 literature values were uncovered. Thus, solubility values were acquired for 89 compounds from the low-variance set and 26 compounds from the high-variance set, and the set of predictions assessed over these values.

**Figure 1.**
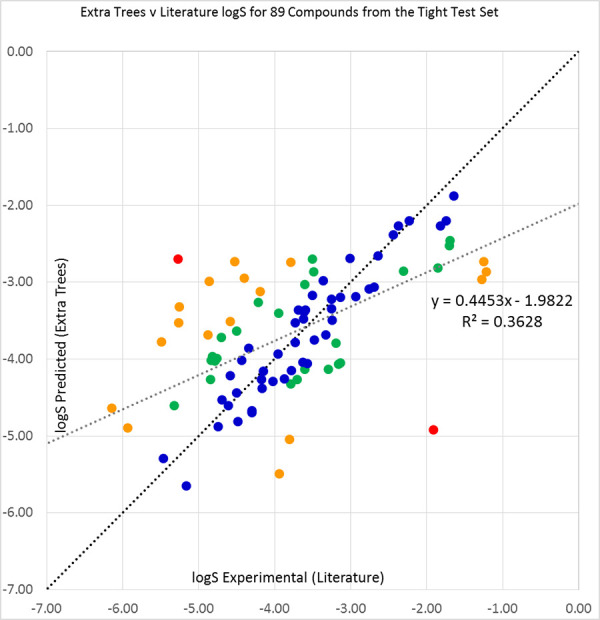
Extra Trees predictions plotted against our sourced literature log S values (see Table A1 for references) for 89 compounds from the 2019 Solubility Challenge tight test set of 100 molecules. Compounds with prediction errors of under 0.5 (blue), 0.5 to 1.0 (green), 1.0 to 2.0 (orange), and over 2.0 log S units (red) are shown in their respective colours. The black diagonal line shows equality of predicted and experimental solubilities, while the grey line is a line of best fit to the data.

**Figure A1.**
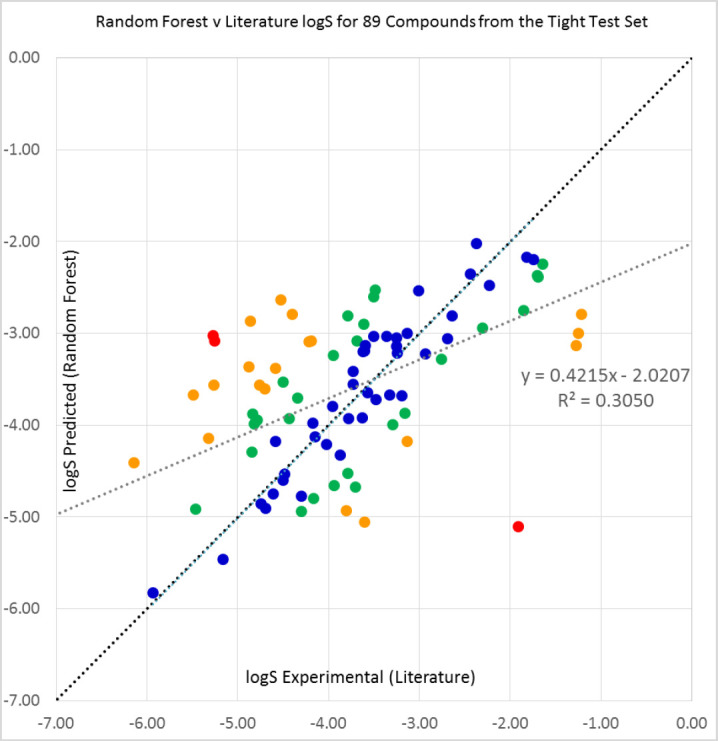
Random Forest predictions plotted against our sourced literature log *S* values (see Table A1 for references) for 89 compounds from the 2019 Solubility Challenge tight test set of 100 molecules. Compounds with prediction errors of under 0.5 (blue), 0.5 to 1.0 (green), 1.0 to 2.0 (orange), and over 2.0 logS units (red) are shown in their respective colours. The black diagonal line shows equality of predicted and experimental solubilities, while the grey line is a line of best fit to the data.

**Figure A2.**
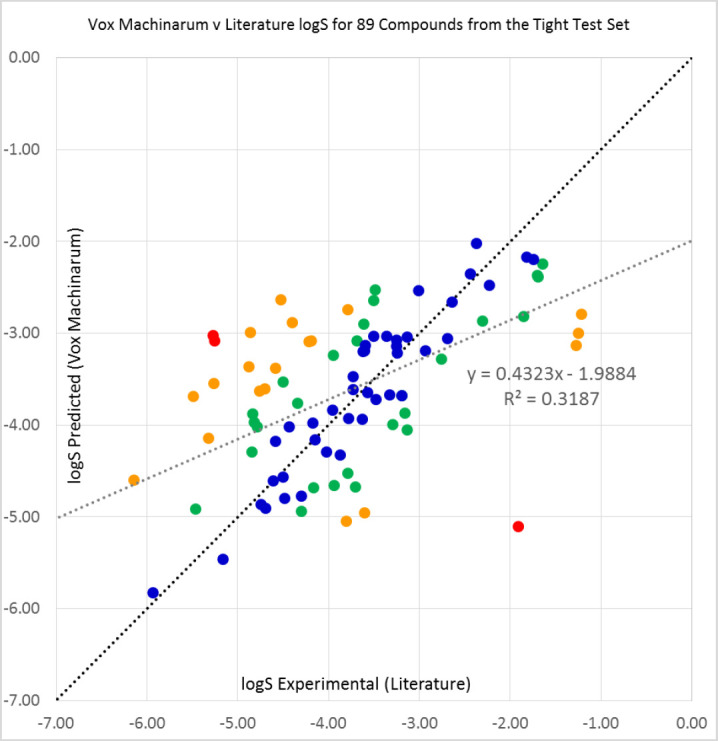
Vox Machinarum predictions plotted against our sourced literature log *S* values (see Table A1 for references) for 89 compounds from the 2019 Solubility Challenge ‘tight’ test set of 100 molecules. Compounds with prediction errors of under 0.5, 0.5 to 1.0, 1.0 to 2.0, and over 2.0 logS units are respectively shown in blue, green, orange and red. The black diagonal line shows equality of predicted and experimental solubilities, while the grey line is a line of best fit to the data.

**Figure A3.**
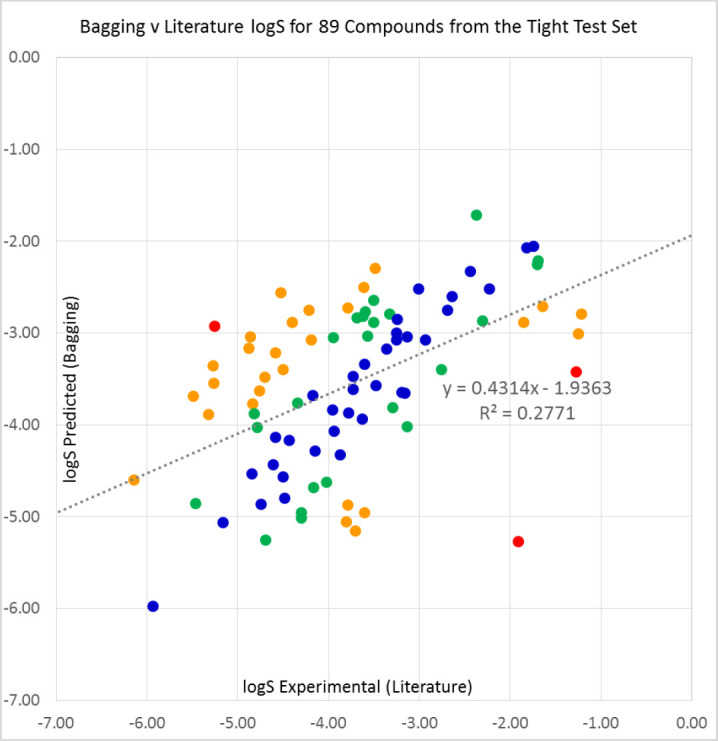
Bagging predictions plotted against our sourced literature log *S* values (see Table A1 for references) for 89 compounds from the 2019 Solubility Challenge tight test set of 100 molecules. Compounds with prediction errors of under 0.5 (blue), 0.5 to 1.0 (green), 1.0 to 2.0 (orange), and over 2.0 logS units (red) are shown in their respective colours. The black diagonal line shows equality of predicted and experimental solubilities, while the grey line is a line of best fit to the data.

For the tight low-variance set, we found that Extra Trees was the most successful method on all five of the criteria shown in Table 2, with an RMSE of 0.897 log *S* units over the 89 compounds for which we had data. Encouragingly, the three methods that had been most successful on the internal validation set, and hence had been submitted to the Challenge, were again the top three ahead of Bagging in this exercise. The predictions are plotted against literature solubilities for Extra Trees in Figure 1, Random Forest in Figure A1, Vox Machinarum in Figure A2, and Bagging in Figure A3.

**Figure 2.**
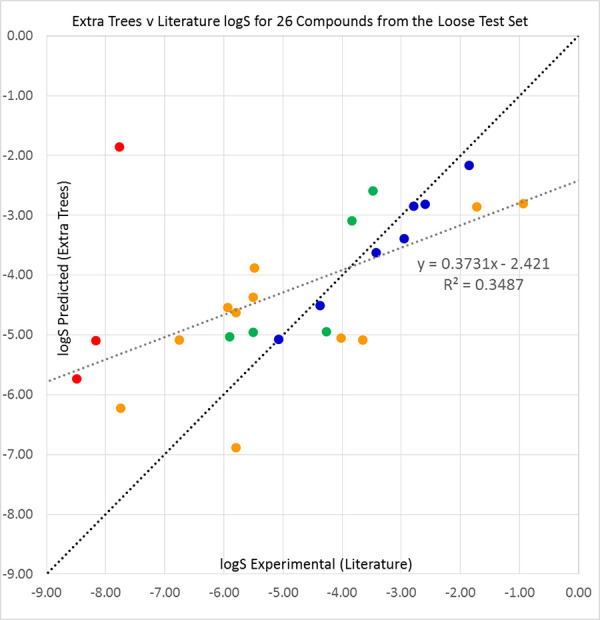
Extra Trees predictions plotted against our sourced literature log S values (see Table A2 for references) for 26 compounds from the 2019 Solubility Challenge loose test set of 32 molecules. Compounds with prediction errors of under 0.5 (blue), 0.5 to 1.0 (green), 1.0 to 2.0 (orange), and over 2.0 log S units (red) are shown in their respective colours. The black diagonal line shows equality of predicted and experimental solubilities, while the grey line is a line of best fit to the data. The large outlier is bisoprolol, as discussed in the text.

**Figure A4.**
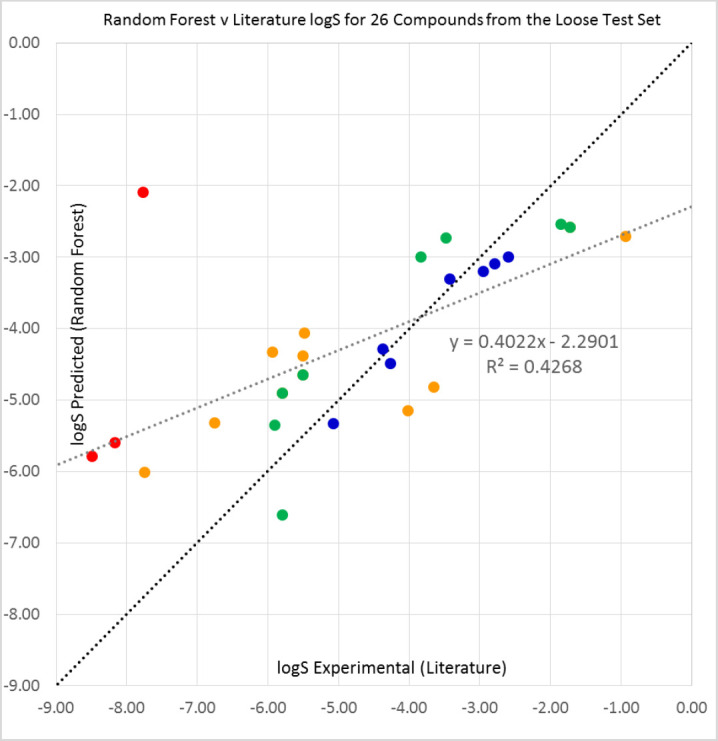
Random Forest predictions plotted against our sourced literature log *S* values (see Table A2 for references) for 26 compounds from the 2019 Solubility Challenge loose test set of 32 molecules. Compounds with prediction errors of under 0.5 (blue), 0.5 to 1.0 (green), 1.0 to 2.0 (orange), and over 2.0 logS units (red) are shown in their respective colours. The black diagonal line shows equality of predicted and experimental solubilities, while the grey line is a line of best fit to the data. The large outlier is bisoprolol, as discussed in the text.

**Figure A5.**
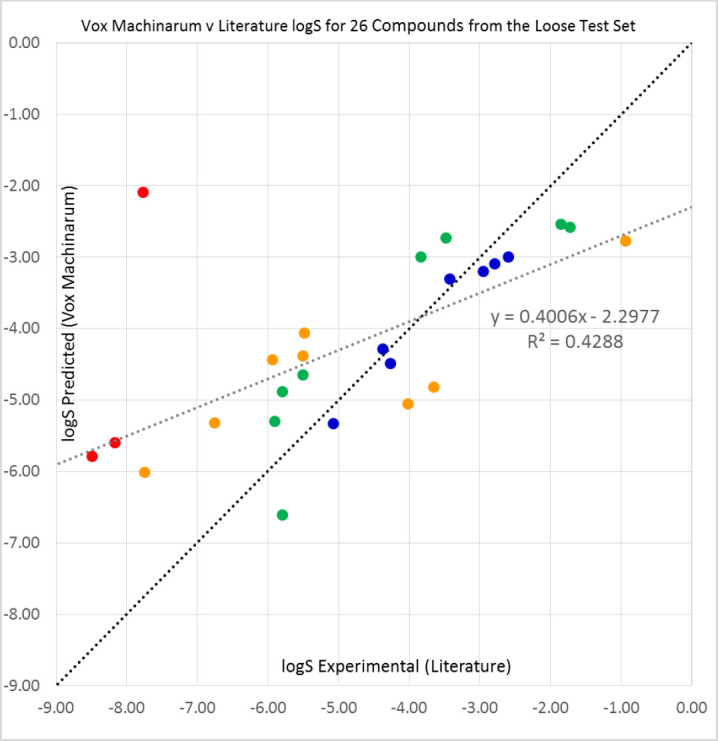
Vox Machinarum predictions plotted against our sourced literature log *S* values (see Table A2 for references) for 26 compounds from the 2019 Solubility Challenge loose test set of 32 molecules. Compounds with prediction errors of under 0.5 (blue), 0.5 to 1.0 (green), 1.0 to 2.0 (orange), and over 2.0 logS units (red) are shown in their respective colours. The black diagonal line shows equality of predicted and experimental solubilities, while the grey line is a line of best fit to the data. The large outlier is bisoprolol, as discussed in the text.

**Figure A6.**
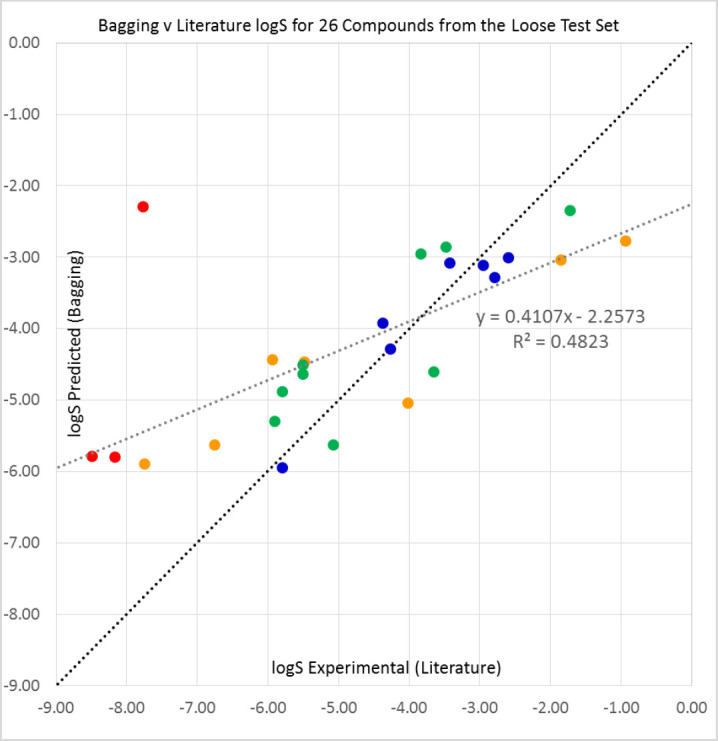
Bagging predictions plotted against our sourced literature log *S* values (see Table A2 for references) for 26 compounds from the 2019 Solubility Challenge loose test set of 32 molecules. Compounds with prediction errors of under 0.5 (blue), 0.5 to 1.0 (green), 1.0 to 2.0 (orange), and over 2.0 logS units (red) are shown in their respective colours. The black diagonal line shows equality of predicted and experimental solubilities, while the grey line is a line of best fit to the data. The large outlier is bisoprolol, as discussed in the text.

For the 26 available compounds from the loose high-variance set, results are shown in Figure 2 for Extra Trees, Figures A4-A6 for other predictors, and Table 3. It is somewhat surprising that Bagging, which we had considered our fourth best predictor, obtained the best results for the loose set. The mutually similar Vox Machinarum and Random Forest classifiers were in a photo-finish for second place and Extra Trees was only fourth best. The RMSE values were notably higher, that is worse, for the loose set than for the tight set, and the proportions of compounds accurately predicted were correspondingly lower.

### 2019 Solubility Challenge: self-assessment on (100 + 32) compounds

Following the completion of this first self-assessment exercise, Avdeef published a paper in *ADMET & DMPK* which revealed the ‘gold standard’ average solubility values for all 132 compounds in the challenge [45]. This facilitated the repetition of the previous self-assessment exercise on the full sets of 100 and 32 compounds.

**Figure 3.**
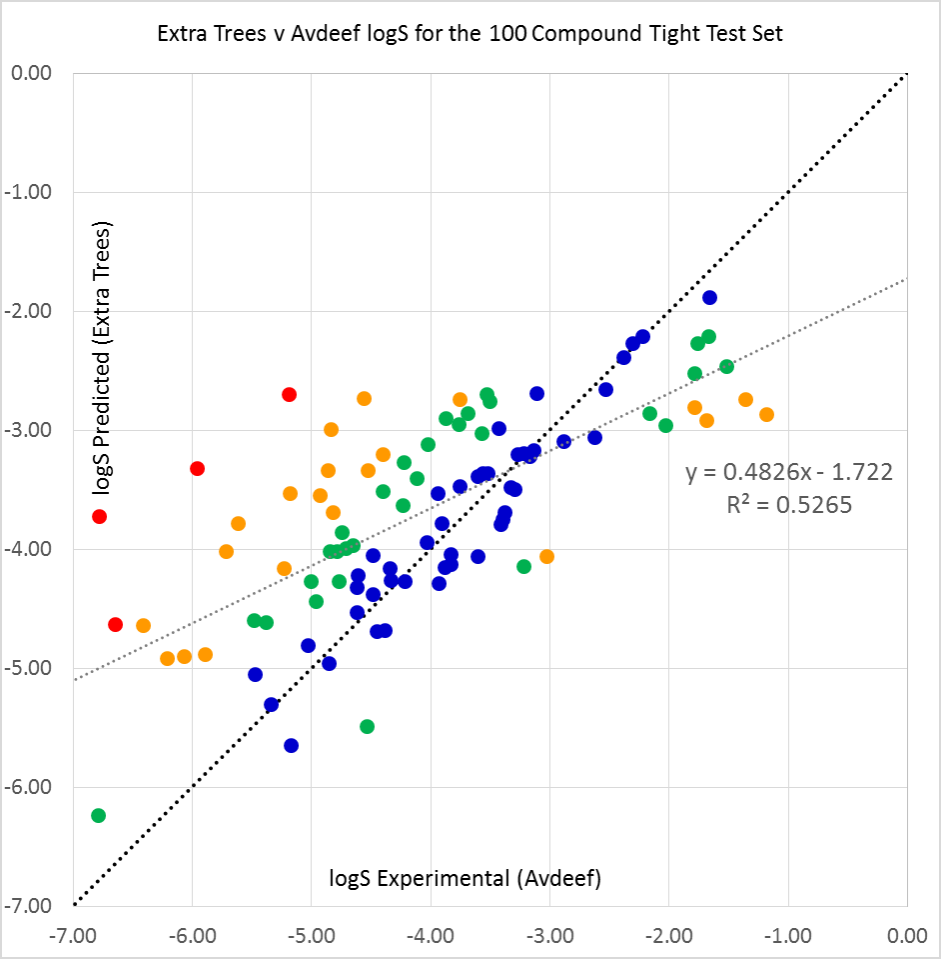
Extra Trees predictions plotted against Avdeef’s average log S values [45] for the 2019 Solubility Challenge tight test set of 100 molecules. Compounds with prediction errors of under 0.5 (blue), 0.5 to 1.0 (green), 1.0 to 2.0 (orange), and over 2.0 log S units (red) are shown in their respective colours. The black diagonal line shows equality of predicted and experimental solubilities, while the grey line is a line of best fit to the data.

**Figure A7.**
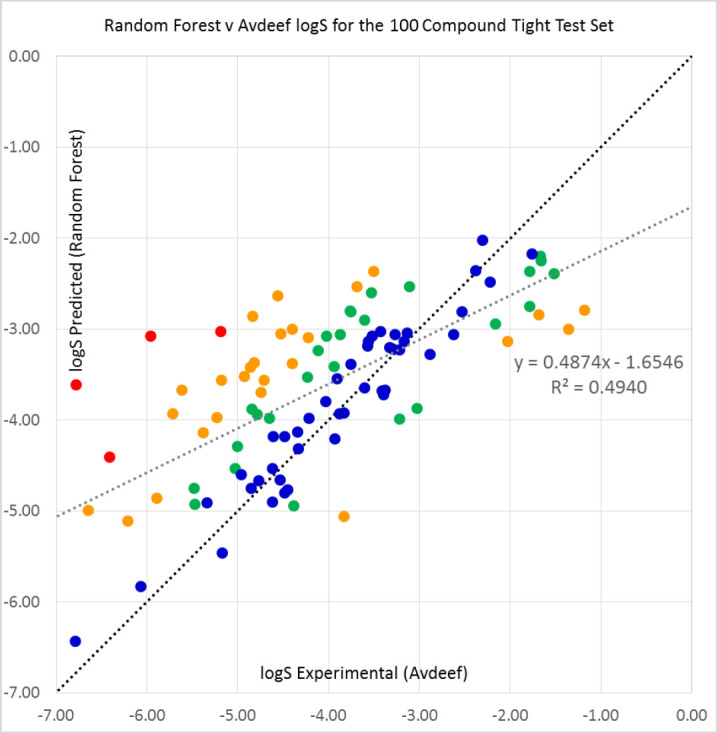
Random Forest predictions plotted against Avdeef’s average log *S* values [45] for the 2019 Solubility Challenge tight test set of 100 molecules. Compounds with prediction errors of under 0.5 (blue), 0.5 to 1.0 (green), 1.0 to 2.0 (orange), and over 2.0 logS units (red) are shown in their respective colours. The black diagonal line shows equality of predicted and experimental solubilities, while the grey line is a line of best fit to the data.

**Figure A8.**
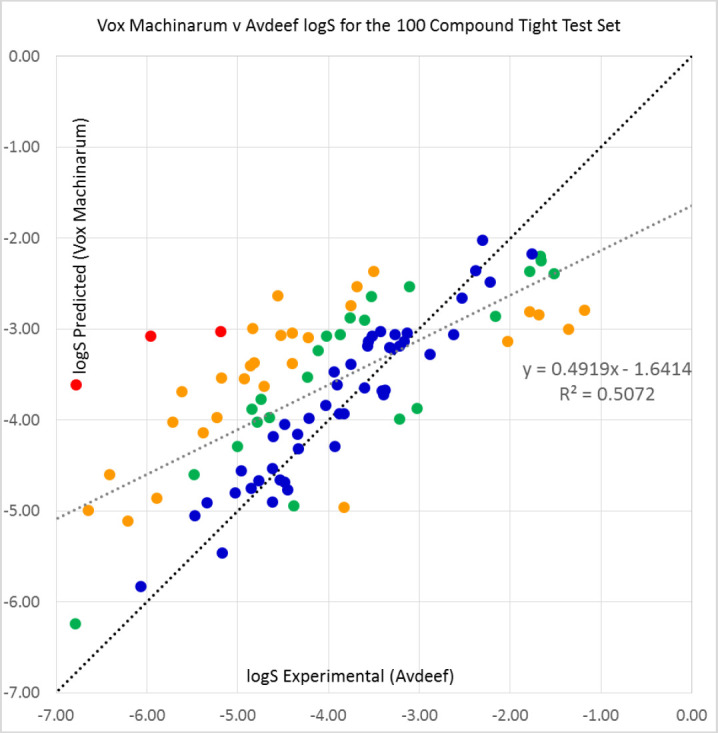
Vox Machinarum predictions plotted against Avdeef’s average log *S* values [45] for the 2019 Solubility Challenge ‘tight’ test set of 100 molecules. Compounds with prediction errors of under 0.5 (blue), 0.5 to 1.0 (green), 1.0 to 2.0 (orange), and over 2.0 logS units (red) are shown in their respective colours. The black diagonal line shows equality of predicted and experimental solubilities, while the grey line is a line of best fit to the data.

**Figure A9.**
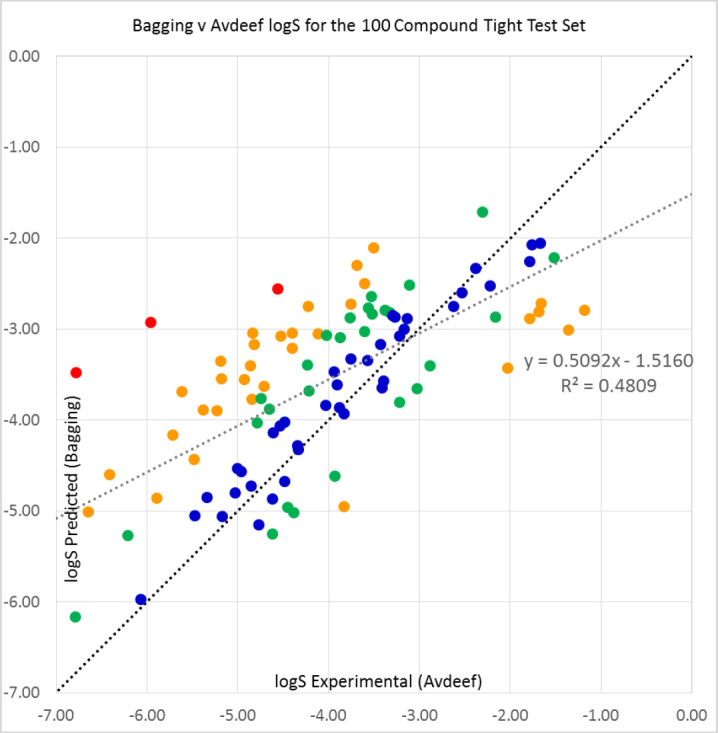
Bagging predictions plotted against Avdeef’s average log *S* values [45] for the 2019 Solubility Challenge tight test set of 100 molecules. Compounds with prediction errors of under 0.5 (blue), 0.5 to 1.0 (green), 1.0 to 2.0 (orange), and over 2.0 logS units (red) are shown in their respective colours. The black diagonal line shows equality of predicted and experimental solubilities, while the grey line is a line of best fit to the data.

For the 100-compound tight set, the relative performances of the classifiers ranked in the same order as they had done previously: Extra Trees first, then Vox Machinarum, Random Forest, and lastly Bagging. However, most measures of classifier quality consistently declined, though the *R*^2^ values were better for Avdeef’s solubilities over 100 compounds than for literature values over 89. For 65 of the 100 compounds, the Random Forest predicted solubility was the second highest of the three individual classifiers, and thus equivalent to the Vox Machinarum median prediction. Results are shown in Table 4 for all classifiers, Figure 3 for Extra Trees, Figure A7 for Random Forest, Figure A8 for Vox Machinarum, and Figure A9 for Bagging.

We noted a significant dependence of outcome on the choice of individual source datapoint for a given compound. For example, the difference in solubilities for tamoxifen between two possible literature sources, log *S* = -8.49 [31] and log *S* = +0.87 [48], is large enough to impact substantially on prediction statistics, and especially so given both the small size of the test set and the larger contribution of compounds with numerically bigger errors to the reported RMSE. Further, the literature value of log *S* = -7.77 [48] for bisoprolol makes it a large outlier, with predicted values all between 1.86 and 2.30; this impression was reinforced by the value of log *S* = 2.09 later given by Avdeef [45] as the average of three experimental determinations. We see no reason to doubt the validity of Avdeef’s number. Since some compounds do indeed have a wide range of reported log *S* values, the effect of any erroneous, or erroneously interpreted or transcribed, datapoints may remain even if averages of experimental values are used. Better approaches may be either to take a median, or else to invest considerable scientific effort in looking at the validity of each experiment, as Avdeef [46] has done, and then picking either a single most trusted datapoint or else an average of only those considered trustworthy. The high level of similarity between the Random Forest and Vox Machinarum results for this test set is a consequence of the frequency with which the Random Forest predictions fall in between those of Extra Trees and Bagging and hence become the median prediction – this occurs for 22 of the 32 compounds.

The majority of the change in prediction quality between those for 89 literature solubilities in Table 2 and for 100 Avdeef solubilities in Table 4 is already seen when the Avdeef solubilities are used for the original 89 compounds, as shown in Table A4. The addition of eleven ‘new’ molecules between Table A4 and Table 4 makes relatively little difference to the prediction quality. Over the 89 compounds, the slight deterioration in RMSE between Tables 2 on the one hand and Tables 4 & A4 on the other is almost exactly in line with the increase in the standard deviation of the experimental log *S* values.

**Figure 4.**
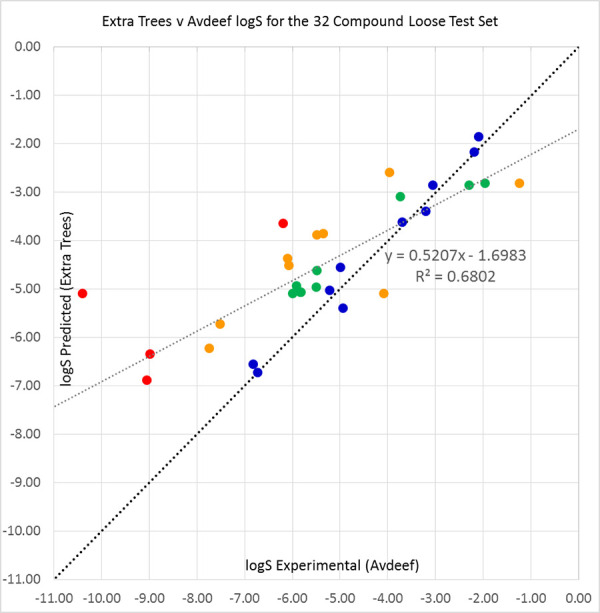
Extra Trees predictions plotted against Avdeef’s average log S values [45] for the 2019 Solubility Challenge loose test set of 32 molecules. Compounds with prediction errors of under 0.5 (blue), 0.5 to 1.0 (green), 1.0 to 2.0 (orange), and over 2.0 logS units (red) are shown in their respective colours. The black diagonal line shows equality of predicted and experimental solubilities, while the grey line is a line of best fit to the data.

**Figure A10.**
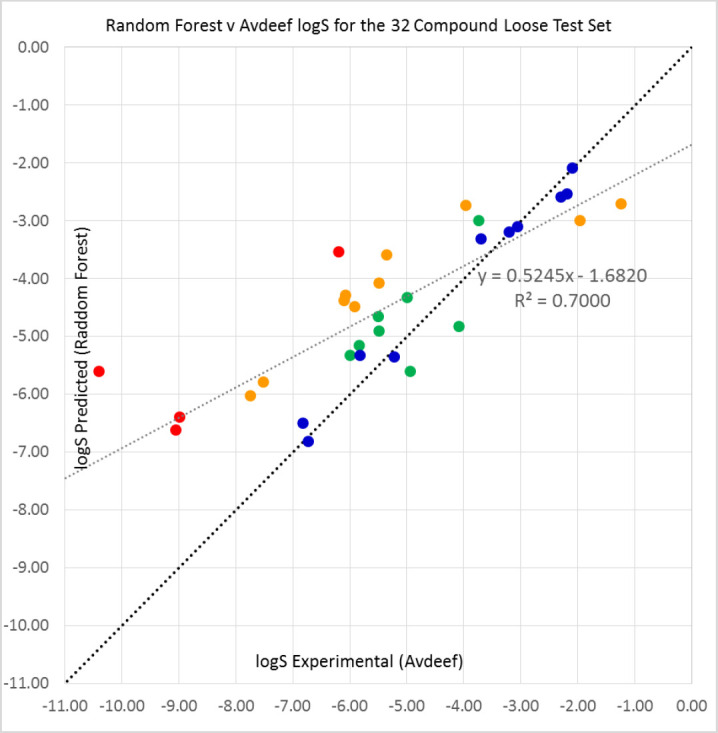
Random Forest predictions plotted against Avdeef’s average log *S* values [45] for the 2019 Solubility Challenge loose test set of 32 molecules. Compounds with prediction errors of under 0.5 (blue), 0.5 to 1.0 (green), 1.0 to 2.0 (orange), and over 2.0 logS units (red) are shown in their respective colours. The black diagonal line shows equality of predicted and experimental solubilities, while the grey line is a line of best fit to the data.

**Figure A11.**
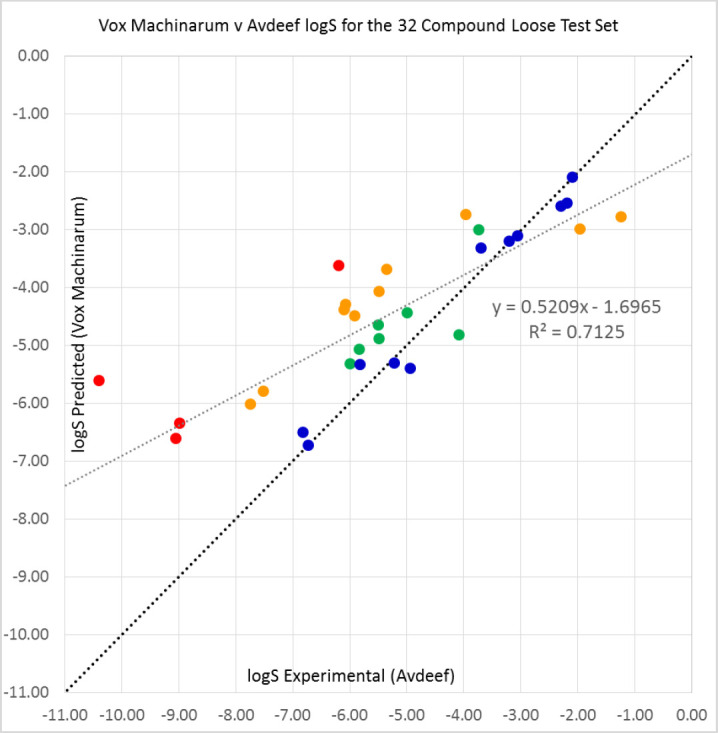
Vox Machinarum predictions plotted against Avdeef’s average log *S* values [45] for the 2019 Solubility Challenge loose test set of 32 molecules. Compounds with prediction errors of under 0.5 (blue), 0.5 to 1.0 (green), 1.0 to 2.0 (orange), and over 2.0 logS units (red) are shown in their respective colours. The black diagonal line shows equality of predicted and experimental solubilities, while the grey line is a line of best fit to the data.

**Figure A12.**
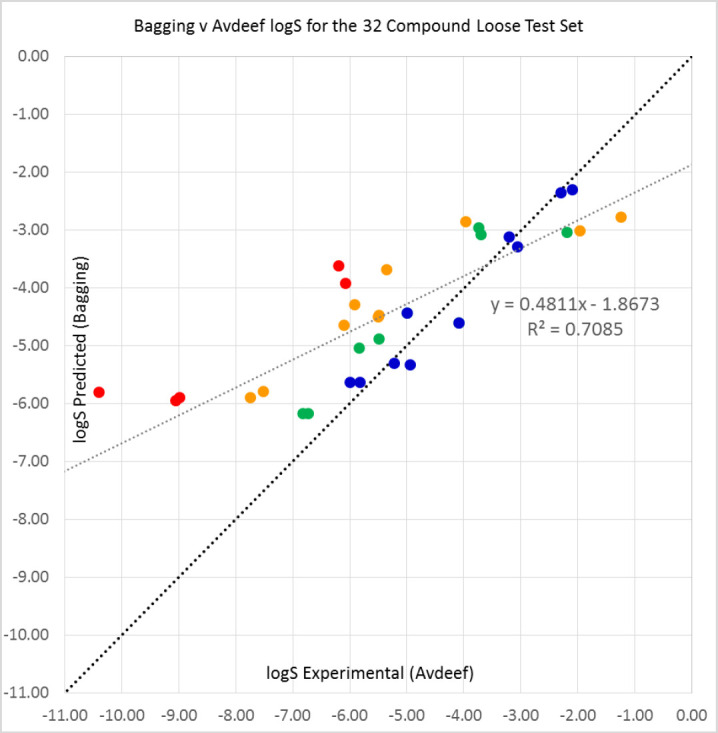
Bagging predictions plotted against Avdeef’s average log *S* values [45] for the 2019 Solubility Challenge loose test set of 32 molecules. Compounds with prediction errors of under 0.5 (blue), 0.5 to 1.0 (green), 1.0 to 2.0 (orange), and over 2.0 logS units (red) are shown in their respective colours. The black diagonal line shows equality of predicted and experimental solubilities, while the grey line is a line of best fit to the data.

Comparisons of predictions for the loose test set of 32 compounds with Avdeef’s data [45] are shown in Table 5, and in Figure 4, Figure A10, Figure A11 and Figure A12 for Extra Trees, Random Forest, Vox Machinarum and Bagging, respectively. Vox Machinarum (RMSE = 1.490) and Random Forest (RMSE = 1.495) classifiers are the two best for this set, and for the reasons discussed previously perform almost identically well. Bagging, which has the best results for the literature solubilities of 26 of these 32 compounds in Table 3, is now the least successful predictor. However, the differences in performance between the predictors here are small and unlikely to be significant. If we compare predictions with the Avdeef solubilities for only those 26 compounds where literature data were also available, Table A5, the performance of the four predictors is almost equal. The modest advantage that Bagging had displayed when using literature solubilities for the same 26 compounds as ground truth disappears when Avdeef solubilities are instead used for comparison.

It is clear from both the 89 v 26 and the 100 v 32 tight versus loose set comparisons that the tight set is better modelled in terms of the error measures such as RMSE, and also the proportions of correct predictions within 0.5 or 1.0 logS units. The increases in RMSE between the 100 and 32 compound tight and loose sets are in fact proportionately smaller than the increases in the standard deviations of the sets themselves, such that the RMSE/SD ratios are marginally smaller for the loose set. Modelling the loose set, however, produces a better *R*^2^. The observation concerning *R*^2^ can be explained by the larger range of extreme solubilities in the loose set, whose maximum and minimum values differ by 9.16 logS units compared with a range of only 5.61 for the tight set.

### Literature vs. Avdeef solubilities for (89 + 26) compounds

**Figure 5.**
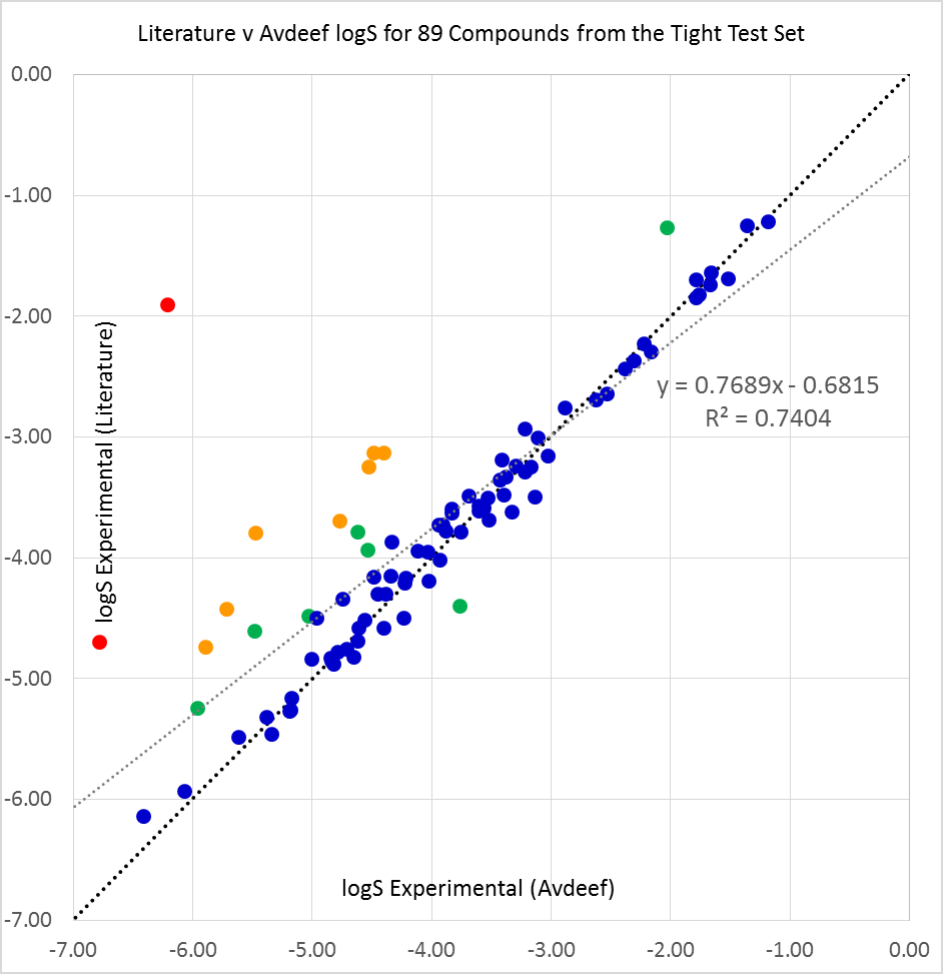
Our sourced literature logS values (see Table A1 for references) plotted against Avdeef’s average log S values [45] for 89 compounds from the 2019 Solubility Challenge tight test set of 100 molecules. Compounds with differences of under 0.5 (blue), 0.5 to 1.0 (green), 1.0 to 2.0 (orange), and over 2.0 logS units (red) are shown in their respective colours. The black diagonal line shows equality of literature and Avdeef solubilities, while the grey line is a line of best fit to the data.

**Figure 6.**
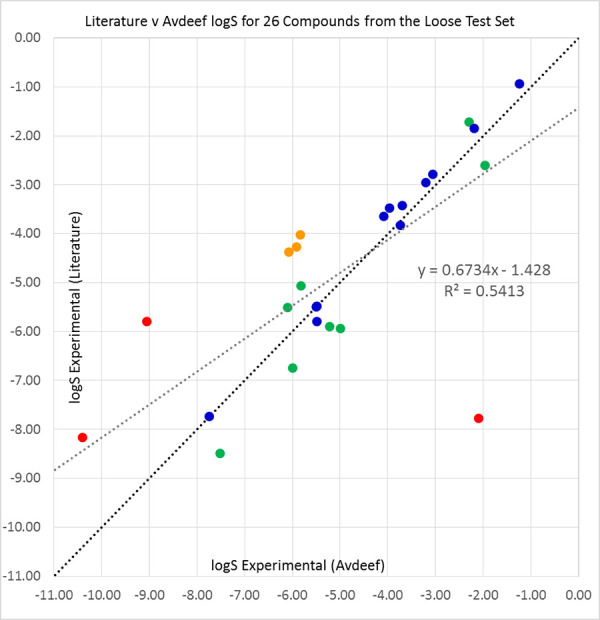
Our sourced literature log S values (see Table A1 for references) plotted against Avdeef’s average log S values [45] for 26 compounds from the 2019 Solubility Challenge loose test set of 32 molecules. Compounds with differences of under 0.5 (blue), 0.5 to 1.0 (green), 1.0 to 2.0 (orange), and over 2.0 logS units (red) are shown in their respective colours. The black diagonal line shows equality of literature and Avdeef solubilities, while the grey line is a line of best fit to the data.

The literature and Avdeef [45] solubilities were compared over the available sets of 89 compounds from the ‘tight’ set and 26 molecules from the loose set. The results are shown in Table 6, and in Figure 5 and Figure 6.

The correspondence between the two sets of experimental solubilities is clearly rather closer than the fits of the models to either sets, comparing Table 6 with Tables 2 and 3. There were nine compounds in the tight set and six in the loose set where the literature and Avdeef log *S* values differed by more than one log *S* unit. For each of these, we checked for errors in our transcription of the literature values into our database and found no such problems. However, we note a wide range of literature values for some molecules. Within the tight set, for example, we found values for griseofulvin of log *S* = -3.25 [47] as used in our literature set and -4.83 [31], a range of 1.58. For haloperidol, the log *S* values of -4.43 [47] from our literature set, -5.26 [48], -5.14 [49] and -5.77 [50], give a range of 1.34. Values of -3.40 and -4.70 for cisapride, both in the collection [48], lead to a log *S* range of 1.30. This contrasts with the average inter-laboratory standard deviation of 0.17 log *S* units that Avdeef obtained for the tight set by careful analysis of published experimental data. [45]

For the loose set, one published value of log *S* = -2.95 for amiodarone [48] is a big outlier and should probably be discounted. The same collection contains the alternative log *S* = -7.17 [48], which is given as an upper bound in [50], while our chosen literature value is log *S* = -8.17 [23]. For clofazimine, reference [48] quotes three values of log *S* of -3.70, -4.68 and -5.68, while our chosen literature value is -5.80 [47]; thus the range is 2.10 log *S* units. Collection [48] gives values of -4.27, -4.09 and -2.48 for saquinavir, the range being 1.79. For each of amiodarone (Avdeef’s log *S* = -10.40), clofazimine (log *S* = -9.05), and saquinavir  (-5.92), Avdeef’s smaller average log *S* [45] values fall outside the range of literature values in our data.

**Figure A13.**
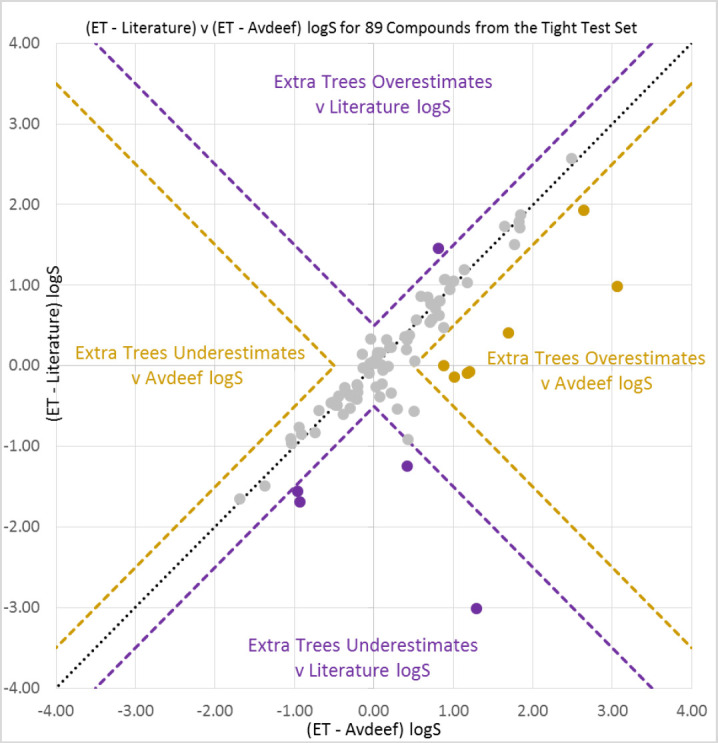
Extra Trees errors obtained when modelling literature solubilities (vertical axis) are plotted against those obtained when modelling Avdeef [45] solubilities (horizontal axis). In the regions delineated by brown lines, Avdeef solubilities are more poorly modelled and literature solubilities better modelled by at least 0.5 logS units (this applies to seven compounds). In the regions delineated by purple lines, literature solubilities are more poorly modelled and Avdeef solubilities are better modelled by at least 0.5 logS units (this applies to five compounds). Thus the Extra Trees modelled values are closer to literature solubilities around 3 o’clock and 9 o’clock on this diagram, whilst Extra Trees is closer to Avdeef solubilities around 12 o’clock and 6 o’clock. These data are for 89 compounds from the tight set.

**Figure A14.**
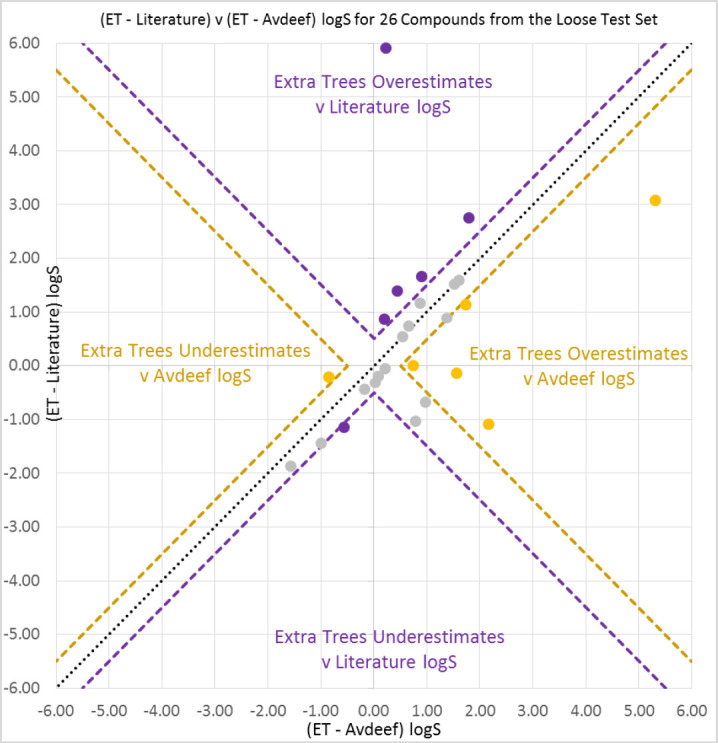
Extra Trees errors obtained when modelling literature solubilities (vertical axis) are plotted against those obtained when modelling Avdeef [45] solubilities (horizontal axis). In the regions delineated by brown lines, Avdeef solubilities are more poorly modelled and literature solubilities better modelled by at least 0.5 logS units (this applies to six compounds). In the regions delineated by purple lines, literature solubilities are more poorly modelled and Avdeef solubilities are better modelled by at least 0.5 logS units (this applies to six compounds). Thus the Extra Trees modelled values are closer to literature solubilities around 3 o’clock and 9 o’clock on this diagram, whilst Extra Trees is closer to Avdeef solubilities around 12 o’clock and 6 o’clock. These data are for 26 compounds from the tight set. The large outlier at the top is bisoprolol.

We have plotted the respective Extra Trees errors when modelling literature solubilities against those obtained when modelling Avdeef solubilities in Figure A13 for 89 compounds from the tight set and Figure A14 for 26 compounds from the loose set. These data show that each set of solubility data is better modelled against either source for similar numbers of compounds. For seven of the 89 compounds the literature solubilities are better modelled by 0.5 log *S* units or more, and for five such compounds Avdeef [45] solubilities are similarly better modelled by at least half a unit. For the 26 compounds in the loose set, six are better modelled against each source of solubilities. However, bisoprolol is a large outlier as discussed above, and use of the Avdeef solubility value seems preferable.

### Possible use of modelling to identify erroneous solubilities

The typical workflow in solubility modelling is to take the experimental solubility data as a gold standard and test computational methods against them. However, in the 2008 Solubility Challenge indomethacin was consistently amongst the worst predicted compounds, with only four of the 99 predictions coming within half a log *S* unit of the ground truth solubility value provided [25]. This led to a re-appraisal of the experimental CheqSol solubility, and to the realisation that indomethacin had in fact hydrolysed under the experimental conditions used. Thus, the solubility value provided was corrected by Comer *et al.* [51] using a revised CheqSol protocol. In this work, only three models all based on similar methodologies and identical descriptors have been used, so the weight of evidence could not approach that of 99 independent predictors. However, when the full results of the 2019 Solubility Challenge are available and analysed, it can be anticipated that any consistently poorly modelled solubilities should be revisited. In the case of bisoprolol, only a single literature value was found in a secondary source for our self-assessment. Clearly, Avdeef’s approach of careful analysis of experimental data would be likely to quickly identify such a value as an outlier.

### Are Avdeef’s solubilities better than literature ones?

Nonetheless, it does not necessarily follow that the carefully curated Avdeef solubilities [45] are clearly better in all respects than literature-harvested ones, especially once clear outliers are identified. In [52] we showed that models trained and tested on supposedly more accurate experimental solubility data were no better than corresponding models based on data harvested from the literature. In the present paper we are comparing only testing on different sets, but again there appears not to be any significant difference in quality between results obtained against largely literature-harvested [30, 47, 48, 53, 54] or against Avdeef’s [45] solubilities. However, around one third of our literature solubilities are from CheqSol experiments [23, 25] originally performed for the 2008 Solubility Challenge and would have been considered part of the accurate set in the context of [52].

We have firstly demonstrated in this paper that models of the tight low-variance set give a substantially better RMSE and more correct predictions than do models of the loose high-variance set, and we secondly note that competently executed models in the literature [3, 4, 15-19, 21-23, 25, 27, 28, 45] typically give RMSE values of between 0.7 and 1.1 log *S* units. We interpret the two observations as indicating that some test sets are harder to model than others. Indeed, we believe that different test sets of compounds can differ substantially in difficulty of prediction. However, the respective comparisons of Avdeef solubilities here and CheqSol solubilities in [52] with literature-harvested data suggest that there is little obvious difference in quality, as expressed by ease-of-modelling, between different solubility compilations covering identical sets of compounds, at least once obviously erroneous or outlying experimental data points are removed.

### How accurate are experimental solubilities?

We might reasonably conceive of error in quoted solubilities as comprised loosely of three components. The first is gross errors, which are errors of kind rather than degree. This could include performing the experiment on the wrong compound, for example due to an unanticipated chemical reaction in the assay, as happened for indomethacin in [25] and was duly corrected in [51]. This might also include typographical errors such as reporting log *S* = -1.74 as log *S* = -7.41, measuring the solubility of the wrong charged form of a compound, wrongly interpreting second hand experimental data, or mistaking kinetic for equilibrium solubilities as discussed in [24]. The second is systematic errors, which might arise between different experimental protocols such as shake flask versus CheqSol, ignoring small inconsistencies in temperature by treating 20 °C, 25 °C and 30 °C as equivalent, measuring solubilities in pure solvent by approximating from different cosolvent concentrations, or accepting data from a slightly wrong pH. The third is random error between different repetitions of the same experimental protocol in the same laboratory, which is claimed to be as low as ±0.05 logS units for CheqSol [23].

Avdeef’s work divided the test solubilities into a tight set (interlaboratory error ±0.17 logS units) and a loose set (±0.62 logS units). The loose set is of essentially the same accuracy as that we envisaged in [52]. The tight set is claimed to be considerably more accurate than that and clearly required considerable effort in its curation. [45, 46] Our results here indeed demonstrate that the tight set generates substantially smaller RMSE values and more correct predictions at both the ±0.5 and ±1.0 logS thresholds than does the loose set. However, our comparison of 89 literature versus Avdeef solubilities for the tight set echoes reference [52] in suggesting that a literature-harvested compilation of solubilities for a given group of compounds does not generate manifestly worse models than does a carefully curated one or a newly consistently measured one. While the respective Avdeef [45] and CheqSol [24-26, 52] solubilities may ultimately prove to be more accurate than literature-harvested sets for the same compounds, it is beyond the power of currently used machine learning modelling methods to demonstrate this unambiguously. What is clear is that Avdeef [45] has at the very least identified a low-variance set of easier-to-model compounds and a high-variance set of harder-to-model molecules.

### How accurate are good predictive solubility models?

As a simple thought experiment, we allow could ourselves to believe that there is ultimately a ground truth intrinsic aqueous solubility for the stablest crystalline polymorph of any given compound. The reported error of a model is its error in reproducing the reported experimental solubilities. This will be some combination of the error of the model in predicting the ground truth, and the error of experiment in matching up to the same ultimate true solubilities. If these errors were hypothetically independent, we would approximately expect the squares of the components to be additive over reasonably large datasets, as with the variances of independent normal distributions or the opposite and adjacent sides of a right-angled triangle. Experience of the field [3, 4, 15-23, 25, 27, 28, 45] suggests that good models typically have RMSEs of 0.7 to 1.1 logS units depending on the difficulty of the test sets, as noted above. If the lower estimate of around 0.17 logS units based on the tight set truly reflects the likely accuracy of good experimental data, then there is considerable remaining scope for models to improve beyond their current level of accuracy. If, however, the accuracy of typical solubility data on which models have been trained and tested is in general closer to the 0.62 logS of the loose set, then existing models have only limited scope for further improvement [28]. Detailed analysis of the results of the 2019 Solubility Challenge should help to resolve this question.

Finally, it is hoped that some of the various first principles methods under development [5-11] are in due course tested on Avdeef’s datasets, or on similar reasonably sized sets. As yet, most have been validated on only a handful of compounds.

## Conclusions

Three Machine Learning models were submitted to the 2019 Solubility Challenge. One was based on Extra Trees, one on Random Forest, and the third was a consensus classifier which we call Vox Machinarum. The results were analysed for the low-variance tight set of 100 compounds and the loose high-variance set of 32 compounds recently published by Avdeef. On the tight set, the Extra Trees method performed best with an RMSE of 0.946 over 100 compounds. For the loose set, the Vox Machinarum (RMSE = 1.490) and Random Forest (RMSE = 1.495) classifiers are best and perform almost equally well.

## Figures and Tables

**Table 1.** Statistical evaluations of ML predictions for the 9 individual predictors and for four possible definitions of the Vox Machinarum classifier over our internal validation set of 36 compounds

Method	RMSE	AAE	*R* ^2^	Err < 0.5	Err < 1.0
Extra Trees	0.766	0.582	0.789	18 (50%)	30 (83%)
Random Forest	0.766	0.607	0.792	17 (47%)	29 (81%)
Bagging	0.827	0.659	0.737	16 (44%)	26 (72%)
MLP	1.017	0.804	0.597	16 (44%)	24 (67%)
kNN man unw k4	1.034	0.792	0.579	18 (50%)	26 (72%)
kNN eu exp k6	1.054	0.818	0.573	14 (39%)	25 (69%)
kNN man exp k4	1.062	0.791	0.548	18 (50%)	25 (69%)
RVM	1.121	0.820	0.509	16 (44%)	25 (69%)
kNN eu unw k6	1.122	0.906	0.528	12 (33%)	22 (61%)
Vox Machinarum (3)	0.760	0.602	0.797	17 (47%)	29 (81%)
Vox Machinarum (5)	0.787	0.627	0.771	17 (47%)	29 (81%)
Vox Machinarum (7)	0.891	0.695	0.695	16 (44%)	29 (81%)
Vox Machinarum (9)	0.944	0.728	0.660	16 (44%)	28 (78%)

**Table 2.** Statistical evaluations of predictions for the Extra Trees, Random Forest and Bagging predictors and for the Vox Machinarum consensus classifier over our sourced literature logS values (see Table A1 for references) for 89 compounds from the 2019 Solubility Challenge tight test set of 100 molecules. The Vox Machinarum predictions reported here were the median of the other three classifiers’ predictions for each compound. The standard deviation of the 89 compounds’ log *S* values is 1.102. Here and in subsequent tables, SD values are calculated using the denominator N for consistency with the definition of RMSE. This is equivalent to calculating the standard deviation of a small set of solubilities rather than using the Bessel correction to emulate the properties of the notional larger distribution from which they might be drawn.

Method	RMSE	RMSE/SD	AAE	*R* ^2^	Err < 0.5	Err < 1.0
Extra Trees	0.897	0.814	0.670	0.363	46 (52%)	70 (79%)
Random Forest	0.958	0.869	0.739	0.305	40 (45%)	67 (75%)
Bagging	1.009	0.915	0.785	0.277	35 (39%)	59 (66%)
Vox Machinarum	0.945	0.858	0.726	0.319	41 (46%)	67 (75%)

**Table 3.** Statistical evaluations of predictions for the Extra Trees, Random Forest and Bagging predictors and for the Vox Machinarum consensus classifier over our sourced literature logS values (see Table A2 for references) for 26 compounds from the 2019 Solubility Challenge loose test set of 32 molecules. The Vox Machinarum predictions reported here were the median of the other three classifiers’ predictions for each compound. The standard deviation of the 26 compounds’ logS values is 2.006.

Method	RMSE	RMSE/SD	AAE	*R* ^2^	Err < 0.5	Err < 1.0
Extra Trees	1.716	0.856	1.226	0.349	7 (27%)	12 (46%)
Random Forest	1.619	0.807	1.160	0.427	7 (27%)	15 (58%)
Bagging	1.558	0.777	1.119	0.482	7 (27%)	16 (62%)
Vox Machinarum	1.617	0.806	1.158	0.429	7 (27%)	15 (58%)

**Table 4.** Statistical evaluations of predictions for the Extra Trees, Random Forest and Bagging predictors and for the Vox Machinarum consensus classifier over Avdeef’s average logS values [45] for all 100 compounds comprising the 2019 Solubility Challenge tight test set of 100 molecules. The Vox Machinarum predictions reported here were the median of the other three classifiers’ predictions for each compound. The standard deviation of the 100 compounds’ logS values was 1.266.

Method	RMSE	RMSE/SD	AAE	*R* ^2^	Err < 0.5	Err < 1.0
Extra Trees	0.946	0.748	0.720	0.527	45 (45%)	75 (75%)
Random Forest	0.989	0.781	0.765	0.494	44 (44%)	70 (70%)
Bagging	1.023	0.808	0.815	0.481	38 (38%)	65 (65%)
Vox Machinarum	0.977	0.771	0.754	0.507	46 (46%)	69 (69%)

**Table 5.** Statistical evaluations of predictions for the Extra Trees, Random Forest and Bagging predictors and for the Vox Machinarum consensus classifier over Avdeef’s average log *S* values [45] for all 32 compounds comprising the 2019 Solubility Challenge loose test set of 32 molecules. The Vox Machinarum predictions reported here were the median of the other three classifiers’ predictions for each compound. The standard deviation of the 32 compounds’ log *S* values was 2.142.

Method	RMSE	RMSE/SD	AAE	*R* ^2^	Err < 0.5	Err < 1.0
Extra Trees	1.517	0.708	1.103	0.680	10 (31%)	19 (59%)
Random Forest	1.495	0.698	1.109	0.700	10 (31%)	18 (56%)
Bagging	1.549	0.723	1.160	0.708	8 (25%)	17 (53%)
Vox Machinarum	1.490	0.696	1.097	0.712	11 (38%)	18 (56%)

**Table 6.** Statistical evaluation of the correspondence between our sourced literature logS values (see Tables A1 and A2 for references) for 89 and 26 compounds respectively from the 2019 Solubility Challenge ‘tight’ and loose test sets against Avdeef’s average log *S* values [45]. The literature solubilities are treated here as ‘predictions’ and Avdeef’s as the gold standard.

Set	RMSE	RMSE/SD	AAE	*R* ^2^	Err < 0.5	Err < 1.0
89 Tight	0.673	0.545	0.331	0.740	73 (82%)	80 (90%)
26 Loose	1.547	0.706	0.963	0.541	12 (46%)	20 (77%)

## Appendix

**Table A1.** Predicted and experimental log *S* values for the low-variance 100-compound test set. **ET**: Extra Trees prediction; **RF**: Random Forest prediction; **BG**: Bagging prediction; **VM**: Vox Machinarum prediction; **LIT**: Literature sourced log *S*; **REF**: Reference for LIT value; **AV**: Avdeef’s average solubility values [45].

Compound	ET	RF	BG	VM	LT	REF	AV
Acetazolamide	-2.39	-2.36	-2.33	-2.36	-2.44	[23]	-2.38
Acetylsalicylic Acid	-2.21	-2.20	-2.06	-2.20	-1.74	[30]	-1.67
Alclofenac	-3.20	-3.00	-3.04	-3.04	-3.13	[30]	-4.40
Ambroxol	-2.90	-3.06	-3.09	-3.06			-3.87
Aripiprazole	-4.63	-4.99	-5.01	-4.99			-6.64
Atovaquone	-4.90	-5.83	-5.98	-5.83	-5.93	[53]	-6.07
Atrazine	-2.86	-2.53	-2.30	-2.53	-3.49	[47]	-3.69
Baclofen	-2.52	-2.37	-2.26	-2.37	-1.70	[47]	-1.78
Barbital,Buta-	-2.21	-2.48	-2.52	-2.48	-2.23	[30]	-2.22
Benzthiazide	-4.02	-3.88	-3.77	-3.88	-4.83	[25]	-4.84
Bromazepam	-3.75	-3.72	-3.57	-3.72	-3.48	[30]	-3.39
Candesartan Cilexetil	-6.24	-6.43	-6.16	-6.24			-6.79
Carbamazepine	-4.14	-3.99	-3.81	-3.99	-3.29	[47]	-3.22
Carbazole	-2.70	-3.03	-3.35	-3.03	-5.27	[53]	-5.19
Carbendazim	-2.73	-2.63	-2.56	-2.63	-4.52	[48]	-4.56
Cefmenoxime	-3.20	-3.06	-2.87	-3.06			-3.27
Cefprozil	-2.92	-2.84	-2.81	-2.84			-1.68
Celecoxib	-4.88	-4.86	-4.86	-4.86	-4.74	[48]	-5.89
Cephradine	-2.87	-2.79	-2.79	-2.79	-1.22	[48]	-1.18
Chlorpropamide	-3.22	-3.14	-3.00	-3.14	-3.25	[23]	-3.17
Cholic Acid,Deoxy-	-4.32	-4.53	-4.87	-4.53	-3.79	[48]	-4.62
Cilostazol	-3.55	-3.52	-3.55	-3.55			-4.93
Cimetidine	-2.46	-2.39	-2.21	-2.39	-1.69	[23]	-1.52
Ciprofloxacin	-3.03	-3.19	-3.34	-3.19	-3.60	[23]	-3.57
Cisapride	-3.72	-3.61	-3.48	-3.61	-4.70	[48]	-6.78
Corticosterone	-3.50	-3.22	-2.85	-3.22	-3.24	[30]	-3.29
Cortisone Acetate	-3.27	-3.09	-2.75	-3.09	-4.21	[30]	-4.22
Cyclosporine A	-4.81	-4.53	-4.80	-4.80	-4.48	[48]	-5.03
Daidzein	-4.16	-3.97	-3.90	-3.97			-5.23
Desipramine	-4.04	-3.92	-3.93	-3.93	-3.63	[23]	-3.83
Dexamethasone	-3.36	-3.14	-2.76	-3.14	-3.59	[30]	-3.56
Diazoxide	-2.98	-3.03	-3.17	-3.03	-3.36	[23]	-3.43
Diclofenac	-5.30	-4.91	-4.85	-4.91	-5.46	[23]	-5.34
Diflorasone Diacetate	-3.69	-3.37	-3.17	-3.37	-4.88	[48]	-4.82
Difloxacin	-4.13	-5.06	-4.96	-4.96	-3.60	[23]	-3.83
Diltiazem	-4.06	-3.87	-3.65	-3.87	-3.16	[23]	-3.02
Diphenylamine	-2.70	-2.60	-2.64	-2.64	-3.50	[53]	-3.53
DOPA,L-	-2.27	-2.17	-2.07	-2.17	-1.82	[47]	-1.76
Enalapril	-2.74	-3.00	-3.01	-3.00	-1.25	[30]	-1.36
Estradiol,17α-	-4.27	-4.29	-4.53	-4.29	-4.84	[48]	-5.00
Estrone	-4.61	-4.14	-3.89	-4.14	-5.32	[54]	-5.38
Ethoxzolamide	-2.95	-2.80	-2.88	-2.88	-4.40	[48]	-3.76
Etoposide	-4.06	-3.65	-3.03	-3.65	-3.57	[53]	-3.60
Eucalyptol	-1.88	-2.25	-2.71	-2.25	-1.64	[53]	-1.66
Fenbufen	-3.53	-3.56	-3.54	-3.54	-5.26	[30]	-5.18
Flumequine	-3.78	-3.55	-3.61	-3.61	-3.73	[23]	-3.90
Flurbiprofen	-4.16	-4.13	-4.28	-4.16	-4.15	[23]	-4.34
Folic Acid	-3.32	-3.08	-2.93	-3.08	-5.25	[25]	-5.96
Ganciclovir	-2.81	-2.75	-2.89	-2.81	-1.85	[48]	-1.78
Glipizide	-3.78	-3.67	-3.69	-3.69	-5.49	[23]	-5.61
Griseofulvin	-3.34	-3.05	-3.07	-3.07	-3.25	[47]	-4.52
Haloperidol	-4.02	-3.93	-4.17	-4.02	-4.43	[47]	-5.71
Ibrutinib	-4.96	-4.75	-4.73	-4.75			-4.85
Indinavir	-5.49	-4.66	-4.07	-4.66	-3.94	[48]	-4.53
Indomethacin	-4.60	-4.75	-4.44	-4.60	-4.61	[51]	-5.48
Indoprofen	-3.97	-3.98	-3.88	-3.97	-4.82	[30]	-4.65
Ketoconazole	-5.05	-4.93	-5.05	-5.05	-3.80	[30]	-5.47
Maprotiline	-4.53	-4.90	-5.25	-4.90	-4.69	[23]	-4.62
Metolazone	-4.15	-3.93	-3.87	-3.93	-3.78	[53]	-3.88
Nabumetone	-3.51	-3.38	-3.21	-3.38	-4.58	[48]	-4.40
Naproxen	-3.63	-3.53	-3.40	-3.53	-4.50	[23]	-4.23
Nelfinavir	-4.92	-5.11	-5.27	-5.11	-1.91	[48]	-6.21
Nevirapine	-3.79	-3.68	-3.65	-3.68	-3.19	[30]	-3.41
Nifedipine	-3.99	-3.56	-3.63	-3.63	-4.76	[53]	-4.71
Nimesulide	-3.86	-3.70	-3.77	-3.77	-4.34	[48]	-4.74
Norfloxacin	-3.09	-3.28	-3.40	-3.28	-2.76	[23]	-2.88
Nortriptyline	-4.29	-4.21	-4.62	-4.29	-4.02	[23]	-3.93
Noscapine	-4.05	-4.18	-4.02	-4.05	-3.14	[48]	-4.48
Ofloxacin	-2.96	-3.14	-3.43	-3.14	-1.27	[23]	-2.03
Oxazepam	-3.94	-3.80	-3.84	-3.84	-3.95	[30]	-4.03
Oxyphenbutazone	-3.53	-3.41	-3.47	-3.47	-3.73	[53]	-3.94
Papaverine	-4.26	-4.32	-4.33	-4.32	-3.87	[23]	-4.33
Perphenazine	-4.38	-4.80	-4.68	-4.68	-4.16	[47]	-4.48
Phenacetin	-2.27	-2.02	-1.71	-2.02	-2.37	[47]	-2.30
Phenazopyridine	-3.12	-3.08	-3.07	-3.08	-4.19	[23]	-4.02
Pindolol	-2.74	-2.81	-2.73	-2.74	-3.79	[23]	-3.75
Pravastatin	-3.34	-3.42	-3.40	-3.40			-4.86
Prednisolone,Methyl-	-3.48	-3.20	-2.82	-3.20	-3.62	[48]	-3.33
Primidone	-2.66	-2.81	-2.60	-2.66	-2.64	[47]	-2.53
Probenecid	-2.99	-2.86	-3.04	-2.99	-4.86	[25]	-4.83
Promazine	-4.69	-4.77	-4.96	-4.77	-4.30	[30]	-4.45
Promethazine	-4.68	-4.94	-5.02	-4.94	-4.30	[30]	-4.38
Repaglinide	-4.27	-4.67	-5.16	-4.67	-3.70	[48]	-4.77
Resveratrol,trans-	-3.47	-3.39	-3.33	-3.39			-3.75
Ritonavir	-5.65	-5.46	-5.06	-5.46	-5.16	[48]	-5.17
Rofecoxib	-4.22	-4.18	-4.14	-4.18	-4.58	[48]	-4.61
Spironolactone	-4.27	-3.98	-3.68	-3.98	-4.17	[47]	-4.21
Strychnine	-3.69	-3.67	-2.79	-3.67	-3.33	[47]	-3.38
Sulfasalazine	-4.64	-4.41	-4.60	-4.60	-6.14	[23]	-6.41
Sulfathiazole	-3.06	-3.06	-2.75	-3.06	-2.69	[23]	-2.62
Sulfisomidine	-2.86	-2.94	-2.86	-2.86	-2.30	[48]	-2.16
Sulfisoxazole	-3.17	-3.04	-2.89	-3.04	-3.50	[48]	-3.13
Sulindac	-4.44	-4.60	-4.56	-4.56	-4.50	[23]	-4.96
Tetracaine	-2.69	-2.53	-2.52	-2.53	-3.01	[23]	-3.11
Tetracycline	-3.19	-3.23	-3.08	-3.19	-2.93	[23]	-3.22
Thiacetazone	-2.76	-2.37	-2.11	-2.37			-3.50
Triamcinolone	-3.36	-3.08	-2.84	-3.08	-3.69	[30]	-3.52
Triamterene	-3.40	-3.24	-3.05	-3.24	-3.95	[47]	-4.11
Warfarin	-4.02	-3.94	-4.03	-4.02	-4.78	[23]	-4.78
Xanthine	-3.39	-2.90	-2.50	-2.90	-3.61	[48]	-3.60

**Table A2.** Predicted and experimental log *S* values for the high-variance 32-compound test set. **ET**: Extra Trees prediction; **RF**: Random Forest prediction; **BG**: Bagging prediction; **VM**: Vox Machinarum prediction; **LIT**: Literature sourced log *S*; **REF**: Reference for LIT value; **AV**: Avdeef’s average solubility values [45]

Compound	ET	RF	BG	VM	LT	REF	AV
Amantadine	-2.17	-2.54	-3.04	-2.54	-1.85	[23]	-2.19
Amiodarone	-5.09	-5.60	-5.80	-5.60	-8.17	[23]	-10.40
Amodiaquine	-4.62	-4.90	-4.88	-4.88	-5.79	[23]	-5.49
Bisoprolol	-1.86	-2.09	-2.30	-2.09	-7.77	[48]	-2.09
Bromocriptine	-4.96	-4.65	-4.50	-4.65	-5.50	[48]	-5.50
Buprenorphine	-4.51	-4.29	-3.92	-4.29	-4.37	[30]	-6.07
Chlorprothixene	-5.09	-5.32	-5.63	-5.32	-6.75	[23]	-5.99
Clofazimine	-6.88	-6.61	-5.95	-6.61	-5.80	[47]	-9.05
Curcumin	-3.85	-3.59	-3.68	-3.68			-5.36
Danazol	-4.37	-4.38	-4.64	-4.38	-5.51	[53]	-6.10
Didanosine	-2.81	-2.71	-2.78	-2.78	-0.94	[47]	-1.24
Diflunisal	-4.55	-4.33	-4.44	-4.44	-5.94	[25]	-4.99
Diphenhydramine	-3.39	-3.20	-3.12	-3.20	-2.95	[23]	-3.21
Etoxadrol	-2.81	-2.99	-3.01	-2.99	-2.60	[48]	-1.96
Ezetimibe	-5.40	-5.60	-5.33	-5.40			-4.94
Fentiazac	-5.06	-5.15	-5.04	-5.06	-4.02	[48]	-5.84
Iopanoic Acid	-3.88	-4.07	-4.47	-4.07	-5.48	[47]	-5.49
Itraconazole	-6.34	-6.39	-5.90	-6.34			-8.98
Miconazole	-5.07	-5.33	-5.63	-5.33	-5.07	[23]	-5.82
Mifepristone	-5.03	-5.35	-5.30	-5.30	-5.90	[50]	-5.22
Omeprazole	-3.62	-3.31	-3.08	-3.31	-3.42	[30]	-3.70
Pioglitazone	-3.65	-3.54	-3.62	-3.62			-6.20
Procaine	-2.86	-2.59	-2.35	-2.59	-1.72	[23]	-2.30
Quinine	-2.85	-3.10	-3.29	-3.10	-2.79	[23]	-3.06
Raloxifene	-6.56	-6.50	-6.17	-6.50			-6.82
Rifabutin	-5.09	-4.82	-4.61	-4.82	-3.65	[48]	-4.09
Saquinavir	-4.94	-4.49	-4.29	-4.49	-4.27	[48]	-5.92
Sulfadimethoxine	-3.09	-3.00	-2.96	-3.00	-3.83	[48]	-3.74
Tamoxifen	-5.73	-5.79	-5.79	-5.79	-8.49	[31]	-7.52
Telmisartan	-6.73	-6.82	-6.17	-6.73			-6.73
Terfenadine	-6.23	-6.02	-5.90	-6.02	-7.74	[25]	-7.74
Thiabendazole	-2.59	-2.73	-2.86	-2.73	-3.48	[25]	-3.97

**Table A3.** Measures of Descriptor Importance. The randomForest package [37] was used to measure the importance of the 173 descriptors based on their individual effects on Mean Squared Error (MSE) for out-of-bag predictions of the training set, and on Node Purity. We also rank the descriptors according to their *R*^2^ measure of correlation with the training set log *S* values. Any descriptor in the top 35 based on MSE or in the top 10 for another measure is listed. Definitions of descriptors are available from reference [55]

	%IncMSE	Rank MSE	IncNodePurity	Rank Pure	*R*^2^ v log *S*	Rank *R*^2^
XLogP	20.95	1	52.36	1	0.47	1
ALogP	14.92	2	23.29	3	0.26	25
ALogp2	14.85	3	26.79	2	0.40	3
MDEC-23	9.23	4	18.09	4	0.42	2
VCH-7	7.23	5	5.40	5	0.06	84
ATSc3	7.03	6	3.42	14	0.03	94
SPC-6	6.44	7	4.04	11	0.17	54
SPC-4	6.30	8	2.85	20	0.13	66
MDEC-33	6.29	9	4.87	8	0.11	69
TopoPSA	5.98	10	1.38	57	0.02	102
VP-2	5.66	11	1.78	47	0.20	45
khs.aaCH	5.30	12	4.62	9	0.21	43
ATSm3	5.29	13	0.94	72	0.18	52
ATSp4	5.26	14	3.46	13	0.29	19
VC-5	5.26	15	2.00	33	0.08	79
C2SP2	5.23	16	4.99	7	0.32	8
SP-6	5.20	17	2.60	23	0.30	11
SC-5	5.03	18	1.90	39	0.06	83
ATSm4	4.96	19	2.47	26	0.21	40
VPC-4	4.93	20	1.23	63	0.13	64
ATSp2	4.60	21	1.49	56	0.30	13
ATSm5	4.53	22	3.79	12	0.21	41
SC-3	4.51	23	0.93	75	0.06	82
naAromAtom	4.47	24	2.87	19	0.27	20
BCUTp-1h	4.42	25	1.99	35	0.25	31
SP-5	4.41	26	3.30	15	0.32	6
SP-4	4.40	27	1.65	51	0.30	17
khs.aasN	4.37	28	1.82	43	0.05	90
ATSm1	4.34	29	1.35	58	0.05	91
SP-7	4.33	30	2.51	25	0.30	12
VP-6	4.26	31	1.96	36	0.22	39
MW	4.21	32	2.07	31	0.20	44
VPC-6	4.19	33	3.21	16	0.15	61
ATSp3	4.18	34	2.75	21	0.29	18
VP-0	4.17	35	1.88	40	0.22	38
C3SP2	3.72	42	4.20	10	0.37	5
nRings6	3.64	43	5.01	6	0.37	4
WTPT-2	3.04	58	3.19	17	0.32	7
MLogP	1.88	87	2.53	24	0.31	9
VAdjMat	0.38	127	0.27	127	0.30	10

**Table A4.** Statistical evaluations of predictions for the Extra Trees, Random Forest and Bagging predictors and for the Vox Machinarum consensus classifier over Avdeef’s average log *S* values [45] for the 89 compounds from the 2019 Solubility Challenge tight 100-molecule test set where literature solubilities were also available. The Vox Machinarum predictions reported here were the median of the other three classifiers’ predictions for each compound. The standard deviation of the 89 compounds’ log *S* values is 1.233.

Method	RMSE	RMSE/SD	AAE	R^2^	Err < 0.5	Err < 1.0
Extra Trees	0.929	0.753	0.697	0.505	42 (47%)	69 (78%)
Random Forest	0.982	0.796	0.748	0.459	41 (46%)	65 (73%)
Bagging	1.015	0.823	0.796	0.445	35 (39%)	60 (67%)
Vox Machinarum	0.967	0.784	0.734	0.474	43 (48%)	64 (72%)

**Table A5.** Statistical evaluations of predictions for the Extra Trees, Random Forest and Bagging predictors and for the Vox Machinarum consensus classifier over Avdeef’s average log *S* values [45] for the 26 compounds from the 2019 Solubility Challenge loose 32-molecule test set where literature solubilities were also available. The Vox Machinarum predictions reported here were the median of the other three classifiers’ predictions for each compound. The Random Forest and Vox Machinarum predicted solubilities were identical for 21 out of these 26 compounds. The standard deviation of the 26 compounds’ log *S* values is 2.191.

Method	RMSE	RMSE/SD	AAE	R^2^	Err < 0.5	Err < 1.0
Extra Trees	1.488	0.679	1.072	0.734	7 (27%)	16 (62%)
Random Forest	1.441	0.658	1.054	0.787	8 (31%)	15 (58%)
Bagging	1.479	0.675	1.083	0.770	7 (27%)	14 (54%)
Vox Machinarum	1.444	0.659	1.055	0.787	8 (31%)	15 (58%)
